# Explantación múltiple, láser de baja potencia y complejo vitamínico B en el tratamiento de la parestesia y disestesia mandibular: reporte de caso

**DOI:** 10.21142/2523-2754-1304-2025-268

**Published:** 2025-11-08

**Authors:** Julissa Sarai Diaz-Campos, Gabriela Araceli Campos-Hermoza, Rubí Esmeralda Sandoval-García, Giancarlo Pares-Ballasco, Edgard David García-Barandiarán

**Affiliations:** 1 Sociedad Científica de Estudiantes de Odontología, Facultad de Odontología, Universidad Nacional Mayor de San Marcos. Lima, Perú. diazcamposjulissa@gmail.com gabriela.campos1@unmsm.edu.pe Lima Perú diazcamposjulissa@gmail.com gabriela.campos1@unmsm.edu.pe; 2 Bachiller en odontología. esuspares19@hotmail.com esuspares19@hotmail.com; 3 Estudiante de pregrado en odontología. rubi.sandoval1@unmsm.edu.pe rubi.sandoval1@unmsm.edu.pe; 4 Especialista en periodoncia e implantología. edgardgarciabarandiaran@gmail.com edgardgarciabarandiaran@gmail.com; 5 Facultad de Odontología, Universidad Científica del Sur. Lima, Perú. Lima Perú

**Keywords:** trastornos sensoriales, terapia por láser de baja potencia, complejo vitamínico B, implantes dentales, sensation disorders, low-level light therapy, vitamin B complex, dental implants

## Abstract

Las afecciones neurosensoriales (AN) tras la colocación de implantes dentales (ID) en la región posterior mandibular representan una complicación por su proximidad con el nervio dentario inferior (NDI). La parestesia y disestesia son los síntomas más reportados que afectan la calidad de vida y requieren un enfoque terapéutico eficaz. Este reporte de caso describe el manejo de las AN utilizando como coadyuvante una terapia láser (TL) combinada con complejo vitamínico B (CV B). Se presenta una paciente de 57 años con pérdida persistente de sensibilidad y episodios espontáneos de sensación tipo descarga eléctrica tras la instalación de tres ID en el sector posterior derecho mandibular. Clínicamente, se corroboró la ausencia de sensibilidad en los tejidos blandos y dolor 8/10 según la escala visual análoga (EVA) en el fondo de surco. La tomografía evidenció una compresión del NDI por los tres ID. Se diagnosticó parestesia con disestesia secundaria a compresión nerviosa. Bajo consentimiento informado, se procedió a la explantación de los ID seguida de una regeneración ósea guiada y, como coadyuvante, se aplicó TL de 650 nm y 976 nm, además de CV B cada 8 horas por 15 días. En la tercera sesión, la paciente reportó recuperación de la sensibilidad y dolor 0/10 EVA, con lo que concluyó el tratamiento. Posteriormente, la tomografía evidenció neoformación ósea. Este caso sugiere que la combinación de TL de baja potencia con CV B es una alternativa terapéutica coadyuvante para el manejo de disestesia y parestesia tras la explantación de implantes compresivos del NDI.

## Abstract (en)

Neurosensory disorders (ND) following the placement of dental implants (DI) in the posterior mandibular region are a potential complication due to their proximity to the inferior alveolar nerve (IAN). Paresthesia and dysesthesia are the most commonly reported symptoms, affecting quality of life and requiring an effective therapeutic approach.

This case report describes the management of ND using adjunctive laser therapy (LT) combined with B-complex vitamins (B-Vit). A 57-year-old female patient presented with persistent hypoesthesia and spontaneous episodes of electric shock-like sensations after the placement of three dental implants in the posterior right mandibular region. Clinical examination confirmed a lack of soft tissue sensation and pain rated 8/10 on the Visual Analog Scale (VAS) at the depth of the sulcus. Tomographic imaging revealed compression of the IAN by all three DI. A diagnosis of paresthesia with dysesthesia secondary to nerve compression was established. With informed consent, the dental implants were explanted, followed by guided bone regeneration. As adjunctive therapy, low-level laser therapy (LLLT) at wavelengths of 650 nm and 976 nm was applied, along with B-Vit administered every 8 hours for 15 days. By the third session, the patient reported recovered sensation and a pain score of 0/10 on the VAS, leading to the conclusion of treatment. Subsequent tomographic imaging showed the bone neoformation. This case suggests that the combination of LLLT with B-Vit may serve as an adjunctive therapeutic alternative for the management of dysesthesia and paresthesia following the explantation of implants compressing the IAN.

## INTRODUCCIÓN

Las alteraciones sensoriales (AS) representan una complicación en la colocación de implantes dentales (ID), comúnmente en la región posterior mandibular, por la proximidad del nervio dentario inferior (NDI) con los sitios de inserción ^1^^,^^2^. Se presenta en diversas etapas del procedimiento, desde la administración de la anestesia local, la elevación del colgajo, la preparación del sitio de osteotomía hasta la colocación del implante, pero también influyen factores como la variabilidad en las técnicas de inserción del ID, las habilidades quirúrgicas e, incluso, la inflamación posoperatoria que afecte los tejidos circundantes, lo que desencadena una pérdida transitoria o persistente de la sensibilidad ^1^^-^^3^. La prevalencia de AS temporales reportadas es del 0 al 24%, mientras que los cambios permanentes varían entre el 0 y el 11%; siendo la disestesia y la parestesia los síntomas más reportados ^2^^,^^3^. La parestesia es una sensación alterada de hormigueo, cosquilleo o anestesia; mientras que la disestesia se caracteriza por una respuesta sensorial desagradable que se manifiesta como ardor o dolor punzante ^4^.

El manejo terapéutico para restablecer la sensibilidad puede ser quirúrgico o no quirúrgico, según la proximidad del implante al NDI, la gravedad y la duración de la sintomatología ^1^^,^^5^^-^^8^. Entre las opciones quirúrgicas, se incluyen la explantación completa o parcial hasta 2 mm del ID, según el grado de compresión o invasión del canal mandibular ^1^^,^^5^^,^^6^. Además, se han propuesto procedimientos como los injertos nerviosos autólogos y alogénicos, técnicas de deslizamiento nervioso, reparación con sutura simple, soldadura láser, descompresión externa, neurólisis interna, escisión de neuroma y neurorrafia ^6^^-^^8^. Por otro lado, las alternativas no quirúrgicas abarcan farmacoterapia (esteroides, analgésicos, estatinas, hormonas, carnitina, vitaminas, coenzima Q, nimodipino, ozono y antivirales) y la fotobiomodulación (FBM) ^6^^-^^8^. 

Recientemente, la FBM tiene mayor relevancia en el manejo de AS por su capacidad para favorecer la recuperación sensorial y reducir el dolor y la inflamación. Esto se debe a que su mecanismo de acción se vincula con la bioestimulación celular, lo que promueve la producción de ATP y la regeneración axonal ^8^. Estudios previos han referido su eficacia en el tratamiento de disestesia y parestesia provocadas por procedimientos odontológicos como la cirugía de terceras molares, cirugía ortognática e implantes dentales ^8^^-^^14^.

Adicionalmente, la administración de vitaminas del complejo B, en particular B1, B6 y B12, también ha mostrado efectos neuroprotectores y regenerativos en el sistema nervioso periférico, lo que facilita la síntesis de mielina y la reparación de fibras nerviosas lesionadas ^15^. Si bien la terapia láser presenta efectos positivos, la complementación con vitamina B12 puede potenciar su efecto y generar un sinergismo que optimice la regeneración neurosensorial y la reducción del dolor ^16^, como lo reportan en el tratamiento de la parestesia del NDI y el nervio lingual posexodoncia de tercera molar ^17^. 

A pesar de los beneficios reportados, la evidencia sobre su aplicación combinada en el manejo de alteraciones sensoriales inducidas por el daño del NDI sigue siendo limitada. Es fundamental desarrollar protocolos clínicos estandarizados para evaluar su eficacia en la regeneración nerviosa y la reducción de la sintomatología ^8^. Por lo tanto, el objetivo de este reporte de caso fue describir el abordaje terapéutico de una AS manifestada como parestesia y disestesia mediante el tratamiento coadyuvante de terapia láser y complejo vitamínico B posterior a la explantación de implantes dentales que comprimían el NDI.

## REPORTE DE CASO

Paciente femenino de 57 años con antecedentes de diabetes mellitus tipo 2, de 29 años de evolución, actualmente controlada. Al acudir a la consulta, refiere la pérdida de sensibilidad con episodios espontáneos de sensación tipo corriente eléctrica en la región posterior mandibular derecha, además de dolor intermitente exacerbado durante la masticación. La paciente indicó que estos síntomas comenzaron después de realizarse una cirugía de colocación de implantes en esa zona y que se mantienen desde hace un año, por lo que solicitaba el retiro de los ID. 

**Figura 1:**
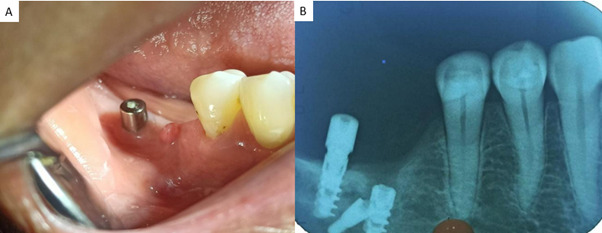
A) Sector posterior derecho mandibular con el pilar del implante dental correspondiente a la pieza 4.7. B) Radiografía periapical donde se observa la presencia de tres implantes dentales.

Durante el examen clínico intraoral, se observó la ausencia de las piezas dentarias 4.6 y 4.7, así como la presencia de un pilar de cicatrización correspondiente al ID en relación con la pieza 4.7. En la evaluación periimplantaria, se registraron profundidades de sondaje de 8 mm en los sitios distal, medio y mesial por vestibular con presencia de sangrado, mientras que por lingual se obtuvieron registros de 5 mm, 5 mm y 6 mm, respectivamente. Adicionalmente, se evidenció una inflamación localizada en al área (figura 1A). La sensibilidad de los tejidos blandos fue evaluada mediante palpación digital y mostró una ausencia de sensibilidad en el área afectada. A nivel de fondo de surco, presentó un edema y dolor a la presión; por ello, se utilizó una escala visual analógica (EVA) para valorar la intensidad del dolor, en la que 0 representa ausencia de dolor y 10 el máximo dolor. La paciente refirió una puntuación EVA 8/10. Inicialmente, la radiografía periapical evidenció la presencia de tres implantes endóseos sin distancia interimplantar en la región correspondiente a las piezas 4.6 y 4.7, con una marcada pérdida ósea vertical en relación con la pieza 4.5 (figura 1B).

**Figura 2:**
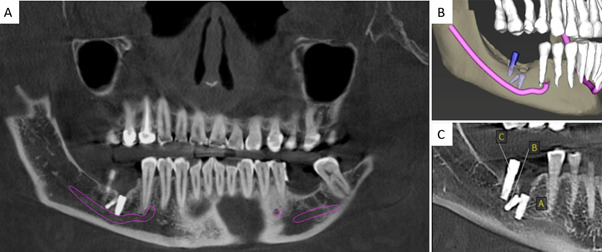
A) Reconstrucción panorámica de tomografía computarizada cone beam. B) Reconstrucción 3D de CBCT con un trazado del nervio dentario inferior. C) Corte sagital de CBCT que evidencia la relación de los implantes dentales (A, B y C) con el nervio dentario inferior.

**Figura 3:**
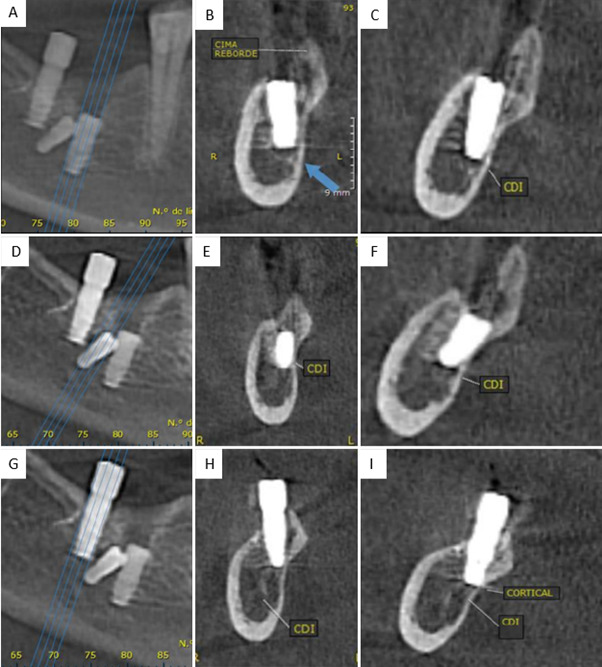
A) Planos 3D de referencia del implante A. B) Corte transversal de implante A. C) Corte coronal de implante B. D) Planos 3D de referencia del implante B. E) Corte transversal de implante B. F) Corte coronal de implante B. G) Planos 3D de referencia del implante C. H) Corte transversal de implante C. I) Corte coronal de implante C.

Para ampliar la evaluación imagenológica, se realizó una tomografía computarizada cone beam (CBCT), la cual confirmó la presencia de tres implantes endóseos con una reabsorción ósea alveolar moderada en sentido horizontal y vertical a nivel vestibular (figuras 2A-2C ). El implante A presentaba una orientación vestibulolingual; el implante B, una inclinación mesioangular en contacto con el implante A, y ambos comprometen el NDI (figuras 3A-3F). Además, el implante C se encontraba sobre el canal mandibular, con proximidad a la cortical lingual del reborde alveolar, y con su porción media y cervical extraóseas (figuras 3G-3I). 

Con base en los hallazgos clínicos y los exámenes auxiliares, se estableció el diagnóstico de parestesia con episodios espontáneos de disestesia asociada a la compresión del NDI por implantes dentales mal posicionados. Previo consentimiento informado, se indicó un tratamiento combinado que incluyó una explantación quirúrgica múltiple de los implantes A, B y C, seguida por la regeneración ósea guiada. Asimismo, se implementó una terapia basada en láser de baja potencia, complementada con un tratamiento farmacológico con Neurobion 5000. Cada gragea contiene 100 mg de nitrato de tiamina (vitamina B1), 100 mg de clorhidrato de piridoxina (vitamina B6) y 5000 mcg de cianocobalamina (vitamina B12).

Siete días antes de la cirugía, se realizó un tratamiento periodontal mediante raspado y alisado radicular, con el objetivo de reducir la carga bacteriana. Como parte del protocolo preoperatorio, la paciente recibió premedicación con amoxicilina 2 g por vía oral, una hora antes del procedimiento. La anestesia local se administró con articaína al 4% con epinefrina 1:100 000 (Artheek®, Laboratorios New Stetic, Colombia), aplicando las técnicas tronculares convencional, troncular alta, así como las técnicas infiltrativas del nervio bucal largo y del nervio lingual.

**Figura 4:**
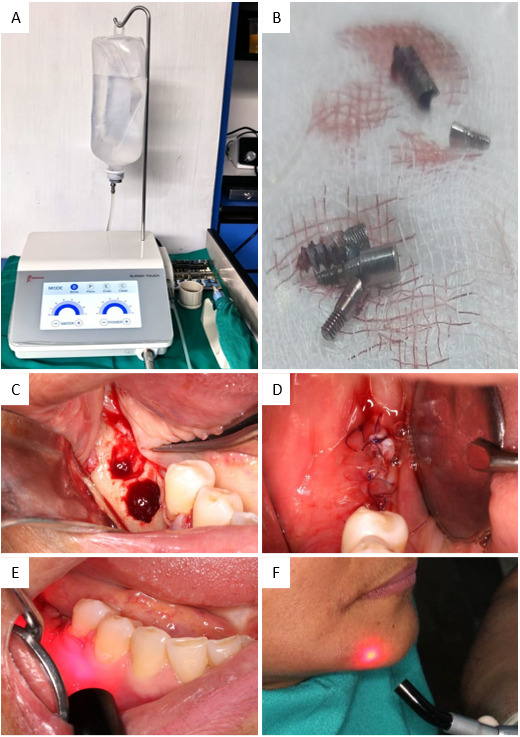
A) Dispositivo piezoeléctrico UDS-P Piezo Ultrasonic Scaler®. B) Implantes removidos quirúrgicamente. C) Fotografía intraoral posterior a la explantación múltiple. D) Cierre de tejidos blandos a través de la sutura en la zona quirúrgica. E) Aplicación de terapia láser intraoral con una longitud de onda de 650 nm. F) Aplicación de terapia láser extraoral con una longitud de onda de 976 nm.

Se realizó una incisión crestal con descarga vestibular a nivel de fondo de surco; posteriormente, se procedió con el levantamiento del colgajo mucoperióstico, seguido por una corticotomía mínimamente invasiva con dispositivo piezoeléctrico UDS-P Piezo Ultrasonic Scaler® (Woodpecker, Guilin, Guangxi, China) (figura 4A), asistido por lupas de magnificación Airx4.5wd 450mm Univet® (Univet Loupes Spa, Italia). Durante la explantación, se mantuvo irrigación continua con suero fisiológico, lo que reduce el riesgo de lesionar el NDI (figuras 4B y 4C). 

Inmediatamente, se realizó la regeneración ósea guiada con hueso bovino particulado liofilizado Bonnefil mix® (Bionnovation Biomedical, Bauru, São Paulo, Brasil) y membrana de colágeno reabsorbible de pericardio 40 mm x 30 mm Surgitime Collagen Pericardium® (Bionnovation Biomedical, Bauru, São Paulo, Brasil). La síntesis de la herida se efectuó con sutura monofilamento reabsorbible de polidioxanona 4/0 DS20 Ethicon® (Somerville, Nueva Jersey, EE. UU.) (figura 4D). 

Posteriormente, se implementó la terapia con láser de diodo LX16 Plus® (Woodpecker, Guilin, Guangxi, China) en el área afectada. A nivel intraoral, se empleó la función prestablecida para terapia cicatrizante (longitud de onda: 650 nm, energía: 3000 J), mediante dos aplicaciones de 60 segundos; y a nivel extraoral se utilizó la función prestablecida de terapia de dolor (longitud de onda: 976 nm, energía: 80J), también con dos aplicaciones de 20 segundos. Ambas se realizaron con movimientos circulares (figuras 4E y 4F). Adicionalmente, se indicó la administración oral de complejo vitamínico B cada ocho horas durante 15 días para optimizar la regeneración nerviosa y aliviar la sintomatología neurosensorial.

Asimismo, se prescribió antibioticoterapia con amoxicilina de 500 mg cada ocho horas durante cinco días, y para la terapia analgésica y antiinflamatoria posoperatoria se indicó clonixinato de lisina 125 mg cada ocho horas y meloxicam 7,5 mg cada 12 horas durante tres días.

En la segunda sesión de terapia con láser, realizada al tercer día posoperatorio, la paciente refirió una notable mejoría de la parestesia y disestesia, sin evidencia de sangrado. A nivel clínico, se detectó una leve tumefacción en la región maseterina y submandibular, por lo que se le indicó que continuara con tratamiento antiinflamatorio durante 48 horas adicionales. Para la tercera sesión de TL, al séptimo día posoperatorio, la paciente no presentó la sintomatología neurosensorial ni tumefacción. La remoción de los puntos de sutura fue realizada sin complicaciones y se recomendó reposo relativo, además de mantener una adecuada higiene oral para favorecer la recuperación. Ante la resolución clínica completa, se le dio el alta a la paciente y se programó una evaluación de control a los seis meses.

**Figura 5:**
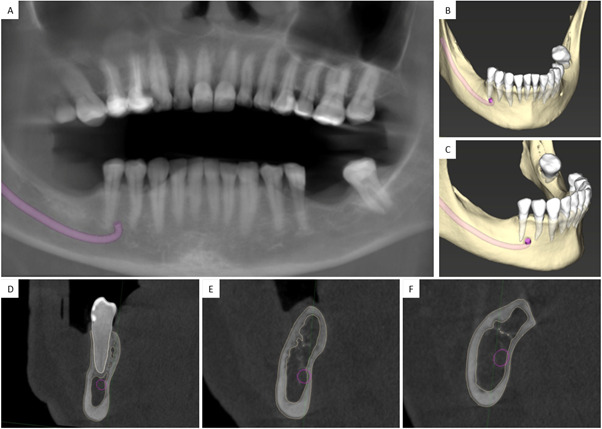
A) Reconstrucción panorámica de CBCT de control. B) Vista frontal de la reconstrucción 3D de CBCT con un trazado del NDI. C) Vista lateral de la reconstrucción 3D de CBCT con un trazado del NDI. D) Corte transversal de la pieza 15 en relación con el nervio dentario inferior. E) Corte transversal. F) Corte coronal de tomografía de control de la zona quirúrgica con adecuada neoformación ósea, sin compromiso del NDI.

En la evaluación clínica de seguimiento, la paciente retornó doce meses después de la operación y se constató la ausencia de sintomatología neurosensorial, con un EVA 0/10, y tras solicitarse una CBCT se evidenció una adecuada neoformación ósea en el sitio intervenido (figuras 5A-5C). Estos hallazgos, junto con la evolución clínica favorable, confirman una respuesta terapéutica satisfactoria al tratamiento quirúrgico complementado con terapia coadyuvante de láser de diodo y la administración oral del complejo vitamínico B. En consecuencia, la paciente fue considerada como clínicamente apta para un nuevo procedimiento quirúrgico implantológico y la posterior rehabilitación protésica en el cuadrante previamente comprometido.

## DISCUSIÓN

Las afecciones neurosensoriales son una consecuencia del daño al NDI, el cual puede presentarse en diversos procedimientos odontológicos, incluyendo tratamientos endodónticos, osteotomía sagital bilateral, exodoncia de terceras molares y cirugía de implantes ^2^^,^^16^. Para la evaluación del compromiso neurosensorial, se emplean pruebas objetivas como la prueba neurosensorial de punción y prueba de discriminación de dos puntos; y pruebas subjetivas, como la escala visual analógica (EVA), que determinan el abordaje terapéutico más adecuado ^13^^,^^18^. 

La elección de la estrategia de tratamiento debe individualizarse según las características clínicas y la evolución del paciente ^1^^,^^7^^,^^8^. Desde el punto de vista fisiopatológico, la lesión del NDI compromete la función mitocondrial, reduce la producción de adenosín trifosfato (ATP) y favorece la despolarización neuronal sostenida, lo que incrementa la excitabilidad nerviosa y contribuye al dolor neuropático. Además, el daño y la inflamación aumentan la producción de especies reactivas de oxígeno (ROS), lo que activa la liberación en cascada de ROS inducida por ROS (RIR), lo que colapsa el potencial de membrana mitocondrial (MMP) y desencadena apoptosis neuronal ^8^. 

Diversos estudios han reportado la efectividad de la terapia de láser en el manejo de alteraciones sensoriales ^7^^-^^9^. Los efectos positivos radican en la optimización de la función mitocondrial a través de la producción de ATP y la estabilización del MMP, de esta forma, reduce la neurodegeneración; además, regula la transmisión sináptica disminuyendo el dolor neuropático y la inflamación ^8^. No obstante, aún no existe un protocolo estandarizado para la FMB en neurorrehabilitación, generando variabilidad en parámetros como longitud de onda, número de sesiones, densidad de energía, potencia y frecuencia ^7^^,^^8^. 

En este estudio se utilizó una longitud de onda de 650 nm con una aplicación de 120 segundos, y se obtuvo una notable disminución de la sintomatología desde la primera aplicación, con mejoría progresiva en controles subsecuentes. Estos hallazgos difieren con los de El Mobadder *et al*. ^10^, quienes reportaron alivio clínico después de 42 sesiones con láser de 635 nm en neuropatías graves del NAI posterior a la exodoncia de terceros molares mandibulares y con Nunes *et al*. ^11^, que emplearon una longitud de onda de 660 nm en 8 sesiones para tratar trastornos neurosensoriales derivadas de procedimientos de rehabilitación oral y cirugía maxilofacial. Asimismo, Ebrahimi *et al*. ^12^ indicaron que un LD de 810 nm mostró mayor eficacia que uno de 980 nm en la reducción de la disestesia en 12 sesiones, mientras que Hakimiha *et al*. ^13^ y Yari *et al*. ^14^ también observaron la recuperación neurosensorial desde la décima sesión en la misma longitud de onda en casos de parestesia derivados de exodoncia de terceras molares y colocación de implantes. Las diferencias en los tiempos de respuesta pueden atribuirse a la complejidad de cada caso, considerando el estado del nervio afectado ^7^, ^8^.

El uso del complejo B ha sido recomendado como terapia coadyuvante en el manejo de las alteraciones neurosensoriales, porque potencia la FBM al mejorar la producción de ATP y proteger las células nerviosas mediante la acción antioxidante de la tiamina (B1). También regula la neurotransmisión con la piridoxina (B6), y la cianocobalamina (B12) favorece la regeneración axonal y la remielinización, lo que acelera la recuperación funcional del NDI ^15^. En casos como el de Da Silva *et al*. ^17^ se reportaron beneficios con el uso de FBM de 808 nm combinada con vitamina B12 en la reducción de parestesia posextracción de terceros molares en un periodo de seis meses, mientras que De Oliveira *et al.*^19^ informaron una efectividad similar entre el uso independiente de LD de 810 nm y etilcobalamina (ETNA, que contiene vitamina B12) en parestesias asociadas a cirugías de implantes y exodoncias de terceros molares inferiores. Sin embargo, la eficacia del complejo B como tratamiento único sigue siendo controversial: en el caso de Mobadder *et al*. ^10^ no observaron mejoría clínica con la administración diaria de vitaminas B1, B6 y B12 dentro de los primeros 21 días posexodoncia, y Ghasemi *et al*. ^20^ tampoco reportaron recuperación neurosensorial con el tratamiento aislado del complejo B (vitamina B1, B2, B6 y nicotinamida) para el abordaje de parestesias posterior a la colocación de implantes dentales.

Estos hallazgos refuerzan la premisa de que la administración exclusiva de vitamina B tiene una efectividad limitada, mientras que su combinación con FBM podría potenciar la recuperación neurosensorial del NDI. No obstante, la respuesta al tratamiento varía según el estado clínico, la duración de la sintomatología y la severidad del compromiso neurológico. 

Este reporte de caso presenta limitaciones. Al referirse a una sola paciente y carecer de grupo de control, no es posible establecer relaciones causales ni generalizar los resultados. La evolución clínica se evaluó mediante parámetros subjetivos, sin la aplicación de pruebas neurosensoriales objetivas estandarizadas. Asimismo, la resolución de la sintomatología referida podría atribuirse con la capacidad individual de recuperación tras la remoción del estímulo compresivo. A pesar de estas limitaciones, no se identificaron reportes similares en la literatura. También se destaca el uso de CBCT tanto para la planificación inicial como para el seguimiento del tratamiento, y se realizó un monitoreo clínico prolongado de la sintomatología, lo que aporta un valor descriptivo al presente informe.

En consecuencia, la implementación de protocolos estandarizados en FBM y su combinación con otras terapias adyuvantes, como el complejo vitamínico B, sigue representando un desafío en la práctica clínica. Por lo tanto, se requieren investigaciones controladas en poblaciones más amplias que permitan validar la efectividad de esta intervención.

## CONCLUSIÓN

La terapia coadyuvante con fotobiomodulación (FBM) y complejo vitamínico B demostró ser una alternativa efectiva para la reducción de la sintomatología sensorial tras la explantación de implantes compresivos sobre el nervio dentario inferior (NDI). 

Si bien la literatura ha reportado el posible rol de cada uno de los tratamientos en la modulación del dolor y en la recuperación funcional del tejido nervioso, este caso clínico refuerza su posible utilidad como tratamiento complementario en casos de parestesia y disestesia secundaria a procedimientos de implantología oral. No obstante, se requieren estudios con un mayor número de pacientes, evaluaciones objetivas y seguimientos prolongados que permitan validar su eficacia y establecer protocolos terapéuticos estandarizados.

## Fuente de financiamiento:

Autofinanciado
